# Polysorbate 21 Can Modulate the Antibacterial Potential of Two Pyrazol Derivatives

**DOI:** 10.3390/biom12121819

**Published:** 2022-12-06

**Authors:** Florin Aonofriesei

**Affiliations:** Department of Natural Sciences, Faculty of Natural and Agricultural Sciences, Ovidius University of Constanta, 1, University Street, 900470 Constanța, Romania; aonofriesei_florin@yahoo.fr

**Keywords:** pyrazole derivatives, polysorbate 21, antibacterial activity, membrane permeability, Gram-positive bacteria, Gram-negative bacteria, crystal violet uptake

## Abstract

The combination of two compounds with known antimicrobial activity may, in some cases, be an effective way to limit the resistance to antibiotics of specific pathogens. Molecules carrying pyrazole moiety are well known for their bioactive properties and have wide applicability in the medical and pharmaceutical field. Surfactants have, among other useful properties, the ability to affect the growth of microorganisms. The paper reports on the effect of the combination of two pyrazole derivatives, (1H-pyrazol-1-yl) methanol 1-hydroxymethylpyrazole (SAM1) and 1,1′methandiylbis (1H–pyrazol) (AM1), with sorbitan monolaurate (polysorbate 21, Tween 21, T21) on the growth of Gram-positive and Gram-negative bacteria. The results demonstrated a different ability of this combination to inhibit *Staphylococcus aureus* and *Escherichia coli*. T21 intensified the inhibitory activity of the pyrazoles to a greater extent in the Gram-negative bacteria compared to the Gram-positive ones, a fact confirmed by time-kill experiments. The experimental data showed that the association of T21 with the pyrazoles led to the increased release of intracellular material and a more intense uptake of crystal violet, which indicates that the potentiation of the antibacterial effect was based on the modification of the normal permeability of bacterial cells. T21 acted as a modulating factor and increased the permeability of the membrane, allowing the accelerated penetration of the pyrazoles inside the bacterial cells. This fact is important in controlling the global increase in microbial resistance to antibiotics and antimicrobials and finding viable solutions to overcome the antibiotic crisis. The paper highlights the possibility of using non-toxic surfactant molecules in antimicrobial combinations with practical applications. This could widen the range of adjuvants in applications which would be useful in the control of resistant microorganisms.

## 1. Introduction

Broadly defined, antimicrobial resistance represents the ability of pathogenic microorganisms to evade and survive when exposed to the action of substances with an antibiotic effect, making the treatment of infections very difficult [1]. This represents a serious challenge for the health care system as it is associated with an increasing risk for vulnerable groups of patients with associated health problems as treatment options are drastically reduced. In the last decades, a huge amount of data has been accumulated regarding the frequency of infections, the health care costs, the morbidity, and the mortality caused by resistant bacteria (Murray et al., 2022). More and more recent reports draw attention to the fact that the number of infections caused by resistant microorganisms is increasing alarmingly worldwide [2,3]. The EARS-NET report [1] shows that in the EU alone more than half of the *E. coli* isolates (53.3%) were resistant to at least one of the major groups of antimicrobials. Moreover, a high percentage (32.1%) of *Pseudomonas aeruginosa* isolates were resistant to one of the antimicrobial groups, and a significant percentage of the strains (19.2%) were resistant to two or more antimicrobial groups. The average presence of methicillin-resistant *Staphylococcus aureus* (MRSA) was 16.4%, while the combined resistance between MRSA and fluoroquinolone was also high (10.4%) [1]. The microbial resistance to antimicrobials is a cause of global concern due to the major impact on public health as well as the increasing costs in the health care system [3]. Worldwide, a very recent report estimates that in 2019 the number of deaths associated with AMR was 4.95 million, the highest number being recorded in the case of six bacteria, a group in which *E. coli*, *Staphylococcus aureus* and *Klebsiella pneumoniae* taken together are directly responsible for 929,000 deaths [2]. The global emergence of pathogen resistance has severely restricted the available treatment choices, and therefore, it has now become a global threat to public health [2,4]. The factors that contribute to the increase in the frequency of AMR are complex and not fully known, but it is believed that they are mainly driven by the use of antimicrobials as well as the transmission of resistant microorganisms between humans and animals and the flow of resistance genes in the natural environment [5]. All these reasons underline the urgent need to find viable solutions to meet the challenge raised by AMR. While finding new compounds with antimicrobial activity for therapeutic purposes has proven to be more difficult and seems to be a longer-term solution, the alternative of using combinations between antimicrobials or between antimicrobials and adjuvants seems more effective in the short term [6].

The increased antibiotic resistance of many pathogens requires scientific efforts to find alternative therapeutic strategies to control infections. This effort is oriented either towards the development of new compounds or towards finding appropriate combinations of already existing compounds with better activity. Pyrazole and its derivatives are valuable for medicinal chemistry because they have a vast potential of biological activities [7,8,9,10] and significant antibacterial potential. There are many reports regarding their inhibitory activity against both Gram-positive and Gram-negative bacteria [11,12,13,14,15]. Some pyrazole derivatives also have significant inhibitory potential against *Mycobacterium* sp., a pathogen that has developed resistance against many antimicrobials in the last decades [16,17,18]. As stated before, one possibility for overcoming the bacterial resistance to antibiotics can be achieved by combining compounds with known activity. The combination is useful when its activity is superior to each component and it is thus able to overcome the pathogen’s resistance. Another way to improve the inhibitory effect of antimicrobials is to use their association with some molecules called adjuvants. They do not have inhibitory activity in themselves, or at most have a reduced activity, but they can decrease the resistance mechanisms, allowing the antimicrobial compounds to block cell growth [6].

The antibacterial effect of a drug depends on the mechanism of action, the target site from a microbial cell, and the interference with other chemical and biochemical compounds. Adjuvants are small molecules with no antimicrobial activity by themselves, or with very low activity when used alone. Associated with antimicrobials in different combinations, they can increase the effectiveness of antimicrobials, especially when the bacteria already show resistance [6]. They fall into two categories depending on the mechanism of action: (i) compounds that act on bacterial target sites and (ii) molecules that increase the host’s defense capacity [6]. There are several mechanisms by which bacteria acquire resistance to antimicrobials [4]: (i) the de novo appearance of mutations [19]; (ii) horizontal gene transfer [20]; (iii) changing the target site for antimicrobials [4]; (iv) reducing the intracellular concentration of the antimicrobial by using efflux pumps [21]; and (v) modification of normal membrane permeability [22]. The last resistance mechanism could theoretically be exploited by using compounds that increase the permeability of the bacterial membranes. Such compounds used in combination with antimicrobials could restrict the growth of pathogens that have developed resistance.

Surfactants are amphiphilic molecules which are able to partition their hydrophilic and hydrophobic groups between different solid and liquid interfaces [23]. In this way, they can alter the surface tension of two liquid media (such as water and oil), and therefore, they are useful as dispersants, emulsifiers, antifoams agents, and lubricants. Surfactants have a wide range of practical applications, such as for food additives, pharmaceuticals, and cosmetics. It has also been shown that their chemical structures could have their own bioactive properties (e.g., antimicrobial, antioxidant, anti-inflammatory, etc.). The antimicrobial properties of surfactants arise from their ability to interact with membrane proteins and lipids [23] and disrupt the metabolism of microbial cells [24]. Most surfactants alter the normal functioning of the inner and outer membranes of pathogens by exploiting their charge and hydrophobicity [25]. Moreover, surfactants can have an important contribution as drug-delivery systems due to their physico-chemical characteristics, thus enhancing the transfer of antimicrobials inside the microbial cell [25]. When used as adjuvants, surfactants can remove resistance to antimicrobials when the resistance mechanism is based on the selective reduction in the membrane permeability of resistant bacteria to specific antimicrobials.

In previous articles, we reported on the antibacterial activity of some pyrazole derivatives, AM1 and SAM1. They seemed promising as inhibitory agents against a significant number of bacteria [26,27]. We found it interesting to explore their effect further by combining the above derivatives with T21. This is a nontoxic, nonionic surfactant containing lauric acid with detergent and emulsifier properties. Although studies have been carried out on numerous molecules with surfactant properties [28], to our knowledge there are no data regarding the association of T21 as an adjuvant in combination with pyrazoles. To understand the effect of combination pyrazoles + T21, we carried out experiments to assess the minimum inhibitory concentration (MIC) and the time-kill characteristics on both Gram-positive and Gram-negative bacteria. Moreover, tests were carried out to identify the changes in the permeability of the bacterial cell membranes under the influence of pyrazoles and their combination with T21. The results indicated an increase in the bactericidal potential of pyrazoles when they were associated with T21 due to the increase in the permeability of the bacterial membranes.

## 2. Materials and Methods

In order to understand the activity of the T21 and pyrazole combination, we used nine clinical and reference bacterial strains, (Table 1), both Gram-positive and Gram-negative.

The experiments were carried out between September 2021 and July 2022. The clinical strains were kindly provided by a medical microbiology laboratory affiliated to Ovidius University of Constanta. The strains were isolated from clinical specimens and identified according to the national approved procedures of the clinical diagnostic laboratories. The strains used in the experiments were chosen to identify the cumulative effect of T21 and the pyrazole derivatives on a variety of opportunistic and pathogenic bacteria, both Gram-positive and Gram-negative, with the aim of having a more comprehensive picture of the effectiveness of the tested compounds. The experiments were carried out in the microbiology laboratory of the Faculty of Natural and Agricultural Sciences, equipped with a safety hood, an autoclave, and the necessary equipment to ensure the safe handling and disposal of hazardous materials.

### 2.1. MIC Evaluation

To establish the MIC value, we used the dilution broth method as described by Hindler et Munro, 2007 [20]. The pyrazole derivatives were diluted in Muller Hinton Broth [MHB-Oxoid (composition, g/L): beef infusion solids, 2.0; casein hydrolysate, 17.5; starch, 1.5; pH = 7.4] at a concentration ranging from 25 to 800 µg/mL. Overnight cultures were grown in MHB, then diluted, and 10 µL was inoculated in tubes containing MHB and pyrazole derivatives to reach a final cell density between 8–9 × 10^5^ and 1 × 10^6^ CFU/mL. Incubation was conducted for 48 h at 37 °C. The MIC endpoint was determined as the lowest concentration of the compound with no visible growth.

### 2.2. Time-Kill Assay

To study the effect the bactericidal activity of SAM1, SAM 2, and their combination with T21, we used the time-kill protocol [29]. Sterile broths containing 0.5 % T21 (w/vol) and pyrazole derivatives at concentrations equivalent to 1, 2, and 4 MIC were prepared. The bacterial culture in early log phase was diluted, and 10 µL was inoculated in tubes containing SAM1, AM1, and their combination with T21 to reach a final concentration of 1 × 10^6^ CFU/mL. At different time intervals (1, 2, 4, 8, and 24 h), 100 µL of culture was serially diluted in MHB and plated out onto Mueller Hinton Agar [MHA-Oxoid (composition, g/L): beef infusion solids, 2.0; casein hydrolysate, 17.5; starch, 1.5; agar, 17; pH = 7.4] in triplicate. The inoculated plates were incubated for 24 h at 37 °C, and the viable colonies were counted. The results from the control and each experimental variant were plotted against time.

### 2.3. Cellular Material Release

The release of cellular material may indicate changes in membrane permeability under the influence of toxic factors. In this regard, we performed evaluations of UV absorbance at 200 nm, 260 nm, and 280 nm in the supernatants of the cultures exposed to T21, SAM1, and T21 + SAM1 by using a slightly changed protocol after Zhang et al., 2017 [30]. Bacterial cultures (*E coli* and *S aureus*) were grown overnight in Tryptone Soy Broth [TSB-Oxoid (composition, g/L): soya peptone, 3 g; glucose, 2.5 g; caseine peptone, 17 g; dipotassium dihydrogen phosphate, 2,5 g; sodium chloride, 5 g; pH = 7.3] and concentrated by centrifugation (10^8^–10^9^ CFU/mL). The cultures were washed three times, suspended in phosphate-buffered saline (PBS) (pH = 7.4), and exposed to T21, SAM1, and T21 + SAM1 for 6 h at 37°C. The controls consisted of bacterial suspensions in PBS without any treatment. After incubation (6 h, 37 °C), the bacterial suspensions were centrifuged (10 min at 14,000 rpm), the cells were separated, and the supernatants were collected. The absorbance of supernatants was measured at 200 nm, 260 nm, and 280 nm by using a double beam Jasco UV-VIS spectrophotometer, and the results were expressed as optical density (OD) values.

The UV absorbance of the compounds alone was recorded at the above wavelengths, and its values were subtracted from the total absorbance of the experimental variants containing the compounds, their combinations, and the bacteria. At the same time, the UV absorbance pattern of the lysates of the *E. coli* and *S. aureus* cultures were used to compare the UV absorbance pattern of the supernatants of the bacterial suspensions exposed to T21, SAM1, and their combination. In order to obtain the cell lysate, the overnight cultures (18–24 h) of the two bacteria (*E. coli* and *S. aureus*) were washed with PBS and suspended in a mixture of 2% SDS + 0.2 N NaOH, followed by incubation at 37 °C for 6 h. The treated cultures were then centrifuged (10 min, 14,000 rpm), the supernatants were collected, and their absorbance was read at 200 nm, 260 nm, and 280 nm. All the experiments were performed in triplicate, with the final data representing the average values.

### 2.4. Uptake of Crystal Violet

The changes in membrane permeability were assessed by crystal violet (CV) assay after Khan et al., 2017 [31] with minor modifications. Overnight cultures of *E. coli* and *S. aureus* were grown in TSB. The cells were harvested at 14,000 rpm for 5 min. The cells were washed twice with sterile water and suspended in PBS (pH = 7.4). T 21, SAM1, and the combination of T21 + SAM1 were added to the bacterial suspension. The cell suspensions were incubated at 37 °C for 6 h. After exposure to the compounds, the cells were separated by centrifugation (at 14,000 rpm for 5 min) and then incubated in PBS containing 10 µg/mL CV for 10 min at 37°C. The cell suspensions were then centrifuged (at 14,000 rpm for 5 min) and the supernatants collected. The absorbance of the supernatants was read on a double-beam Jasco UV-VIS spectrophotometer at 590 nm. Control samples were prepared and incubated similarly without any treatment. In order to highlight the changes over time in the membrane permeability under the influence of the compounds and their combinations, a similar procedure was used as above. At variable time intervals (0, 2 h, 4 h, 8 h, and 24 h), 2 mL of suspension from each of the experimental variants and the controls was taken, centrifuged, and washed with PBS. Then, the suspensions were incubated in the presence of CV for 10 min at 37 °C. They were afterwards centrifuged, the cells were separated, and the absorbance of the supernatants was read at 590 nm. The optical density of the freshly prepared solution containing 10 µg/mL CV used in the assay was to be considered 100%. The amount of crystal violet uptake for all the samples was calculated using the formula [31]: % crystal violet uptake = 100 − [(OD of sample/OD of crystal violet solution) × 100]. Statistical analysis of the data (basic statistics, the Pearson product–moment correlation, and the paired-samples *t* tests were conducted using STW Statistics 18 software.

## 3. Results

### 3.1. MIC Values of Pyrazoles and Their Combination with T21

The inhibitory effect of SAM1 and AM1 varied and depended on the compound and bacterial strains. This was reflected by the fluctuation between the minimum and maximum values in SAM1 (between 50 and 200 µg/mL) and AM1 (between 100 and 400 µg/mL). SAM1 appeared to be more effective in its antibacterial potential than AM1. The former showed a mean inhibitory activity at around 177.7 µg/mL, while the latter had a mean MIC value of 311.1 µg/mL. On average, the MIC value for SAM1 is almost two times lower compared to that recorded for AM1 (Table 2).

The addition of T21 resulted in the obvious decrease in mean MIC values from 177.7 to 55.5 µg/mL for SAM1 µg/mL and from 311.1 to 144.4 µg/mL for AM1, respectively (Table 2). This fact suggested a potentiating effect of the inhibitory activity of approximately three times for SAM1 and two times for AM1, when the two pyrazole derivatives were associated with T21. These observations led us to investigate the influence of T21 through time-kill experiments to find out more details related to the time dynamics activity of these associations.

### 3.2. Time-Kill Dynamics

**Figure 1:**
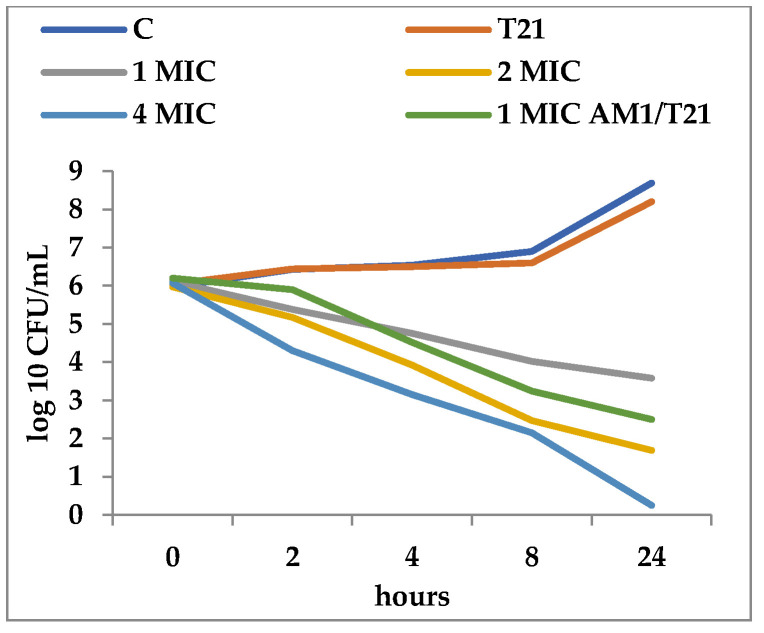
Effect of T21 (0.5%), AM1 (1-4 MIC), and its association with T21 (AM1 1MIC + T21) on the time-killing dynamics in *S. aureus* ATTC 33952.

**Figure 2:**
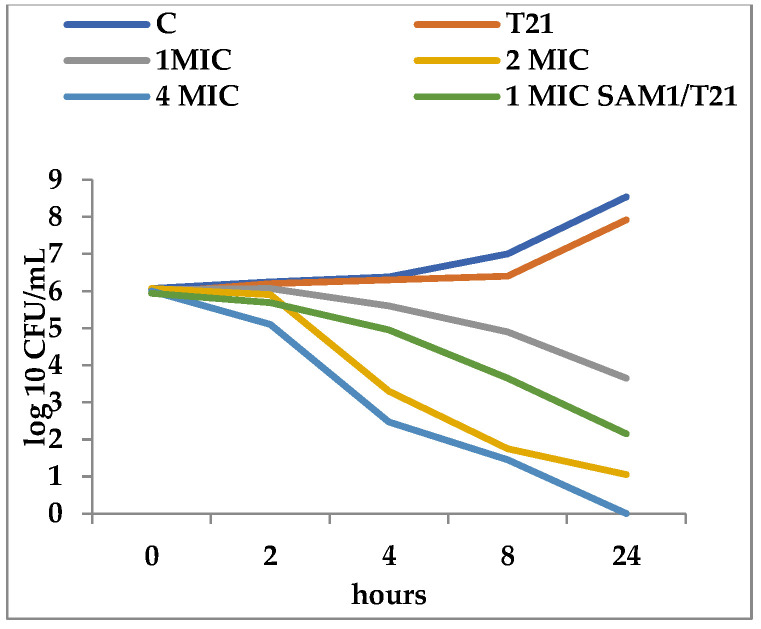
Effect of T21 (0,5%), SAM1 (1-4 MIC, and its association with T21 (SAM1 1MIC + T21) on the time-killing dynamics in *S. aureus* ATCC 33952.

**Figure 3:**
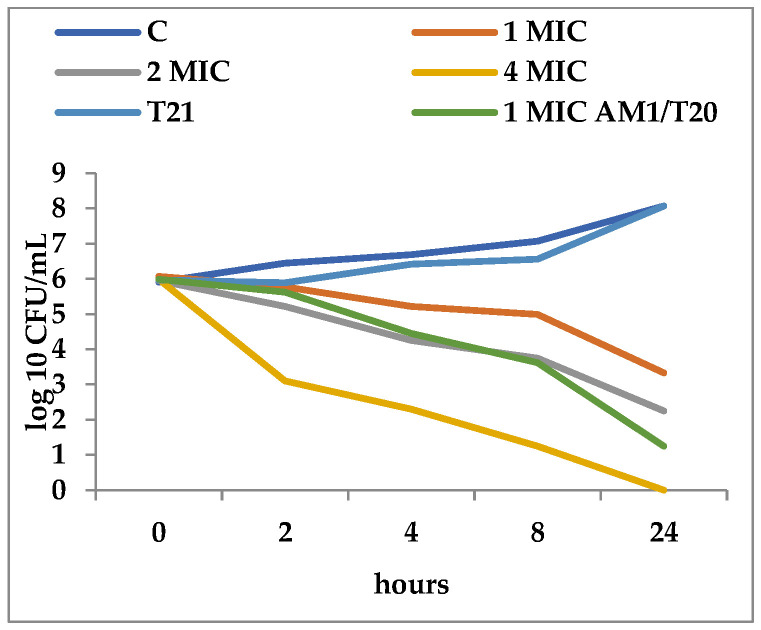
Effect of T21 (0.5%), AM1 (1-4 MIC, and its association with T21 (AM1 1MIC + T21) on the time-killing dynamics in *E. coli* ATCC 11229.

**Figure 4:**
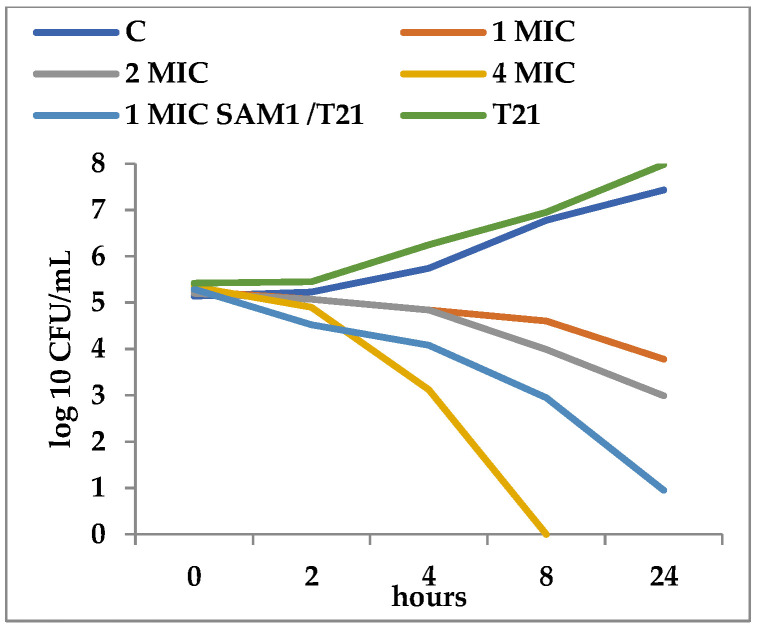
Effect of T21 (0,5%), AM1 (1-4 MIC), and its association with T21 (SAM1 1MIC + T21) on the time-killing dynamics in *E. coli* ATCC 11229.

T21 alone did not show any inhibitory effect in itself on *S. aureus* ATCC 25923 growth, although a slight lag phase could be observed in some cases (Figure 1 and Figure 2). The association of T21 with AM1 and SAM1 at concentrations of 1 MIC resulted in a higher rate of reduction in *S. aureus* ATTC 33952 cell viability than in the case of the pyrazoles used separately (Figure 1 and Figure 2). Minimal differences were recorded between the two pyrazole derivatives, the reduction in cell viability being somewhat higher in the case of the association of T21 with SAM1 than with AM1 (Figure 1 and Figure 2). The number of viable cells was lower at 1 MIC in the presence of T21, after 24 h, compared to the compounds used separately (Figure 1 and Figure 2). When AM1 was used alone, the number of viable *S. aureus* ATTC 33952 cells decreased from 6.11 log10 CFU/mL to 3.58 log10 CFU/mL after 24 h (Figure 1). In contrast, the combination of AM1 + T21 led to a more pronounced decrease in the population of *S. aureus* ATTC 33952 from 6.2 log10 CFU/mL to 2.5 log10 CFU/mL (r = −0.85) after 24 h of incubation (Figure 1). In the case of the SAM1 + T21 combination, the decrease was also substantial, from 5.94 log10 CFU/mL to 2.15 log10 CFU/mL (r = −0.96) and higher than in the case of using SAM1 alone (Figure 2). The association effect of the two pyrazoles with T21 was significantly more consistent in the case of *E. coli* ATCC 11229. The reduction in cell viability at 1 MIC when the compounds were associated with T21 exceeded the time-kill rate recorded at 2 MIC for both AM1 and SAM1 used alone (Figure 3 and Figure 4). The most obvious reduction in viable cells was observed after 24 h in the case of the SAM1 + T21 combination, from 5.29 log10 CFU/mL to 0.95 log10 CFU/mL (r = −0.97) (Figure 4).

These observations support the point of view that T21 can increase the inhibitory potential of the two pyrazoles.

### 3.3. Cellular Material Release

The changes in UV pattern absorbance were recorded for *E. coli* ATCC 11229 and *S. aureus* ATCC 33952 exposed to T21, SAM1, and T21 + SAM1. The association of T21 and SAM1 had different effects on the type of macromolecules released from the cells, as evidenced by the differences in absorbance at the different wavelengths. Moreover, the absorbance varied depending on the bacterial group (Gram-positive or Gram-negative) subjected to the respective compounds.

**Figure 5:**
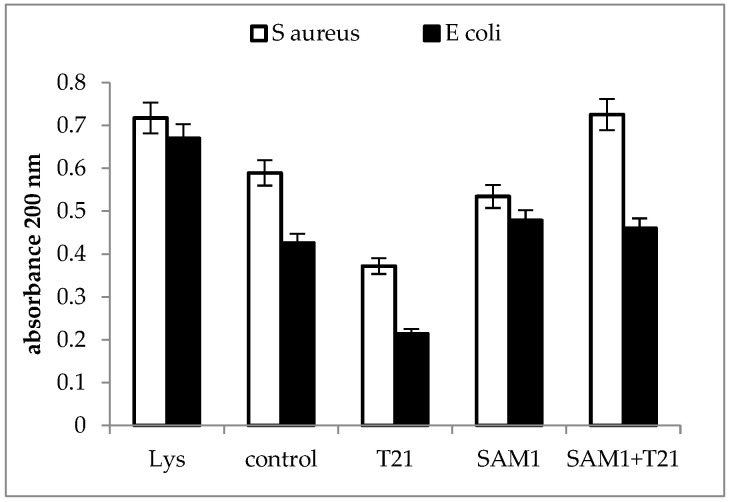
UV absorbance at 200 nm in *S. aureus* ATCC 33952 and *E. coli* ATCC 11229 cultures after treatment with T21, SAM1, and SAM1 + T21 (concentration of compounds and combinations: T21 0.5%; SAM1 1MIC; SAM1 1MIC + T21 0.5%).

A significant increase in absorbance at 200 nm was recorded for the SAM1 + T21 combination in *S. aureus* ATCC 33952, while in *E coli* ATCC 11229 the changes were minimal compared to the control. At the same time, T21 seemed to play a stabilizing and protective role on the *E. coli* ATCC 11229 cells since, apparently, the level of macromolecules with absorbance at 200 nm released outside the cells was lower compared to the control (Figure 5).

**Figure 6:**
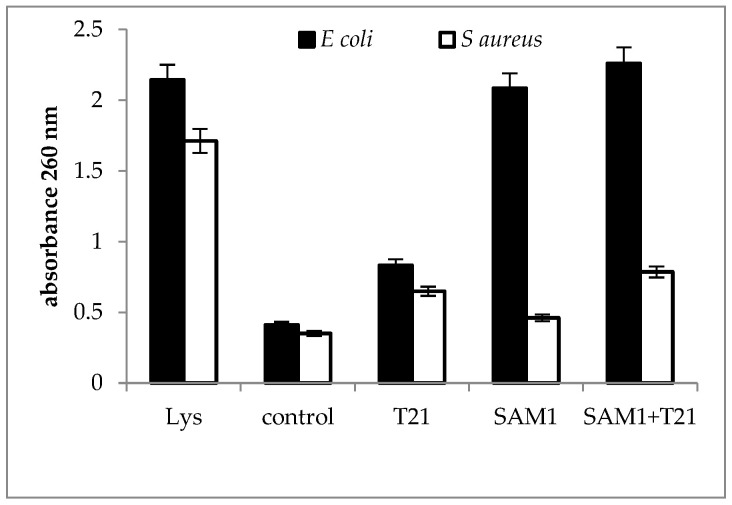
UV absorbance at 260 nm in *S. aureus* ATCC 33952 and *E. coli* ATCC 11229 cultures after treatment with T21, SAM1, and SAM1 + T21 (concentration of compounds and combinations: T21 0.5%; SAM1 1MIC; SAM1 1MIC + T21 0.5%).

A larger difference in absorbance between the two bacteria exposed to SAM1 and its combination with T21 was observed at 260 nm; *E. coli* ATCC 11229 was characterized by high values for both SAM1 and SAM1 + T21, while in *S. aureus* ATCC 33952 the values were much lower for all the experimental variants compared to the controls (Figure 6). In *S. aureus* ATCC 33952, SAM1 causes minimal changes in absorbance, with a slight increase above the control after exposure to T21 and the combination of T21 + SAM1. The release of cellular material was more intense in *E. coli* 11229 when the combination SAM1 + T21 was used and was observed to have a value close to that recorded for the lysed culture. This suggested that T21 could increase the permeability of the cell membrane, which led to the increased release of nucleic acids outside the cells.

**Figure 7:**
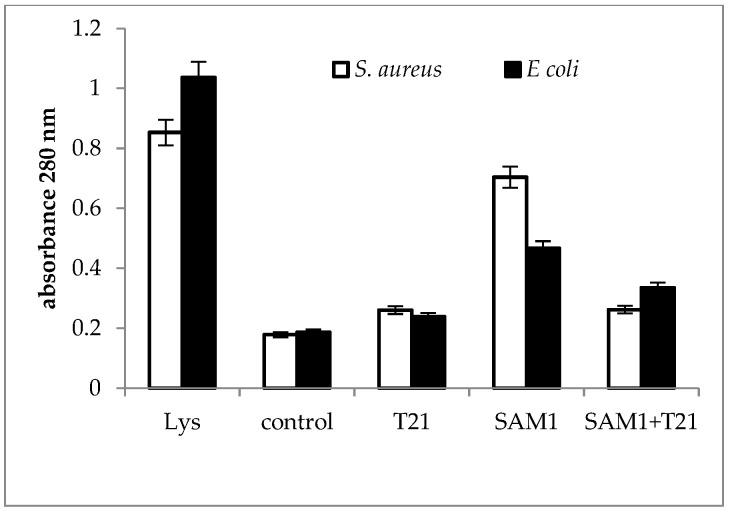
UV absorbance at 280 nm in *S. aureus* ATCC 33952 and *E. coli* ATCC 11229 cultures after treatment with T21, SAM1, and SAM1 + T21 (concentration of compounds and combinations: T21 0.5%; SAM1 1MIC; SAM1 1MIC + T21 0.5%).

The absorbance profile at 280 nm was almost similar for the two bacteria (Figure 7). The cellular protein release was most intense when the bacteria were exposed to SAM1 compared to T21 alone and the T21 + SAM1 combination and suggested that T21 might play a protective role on cell structure and composition.

### 3.4. Crystal Violet Uptake

**Figure 8:**
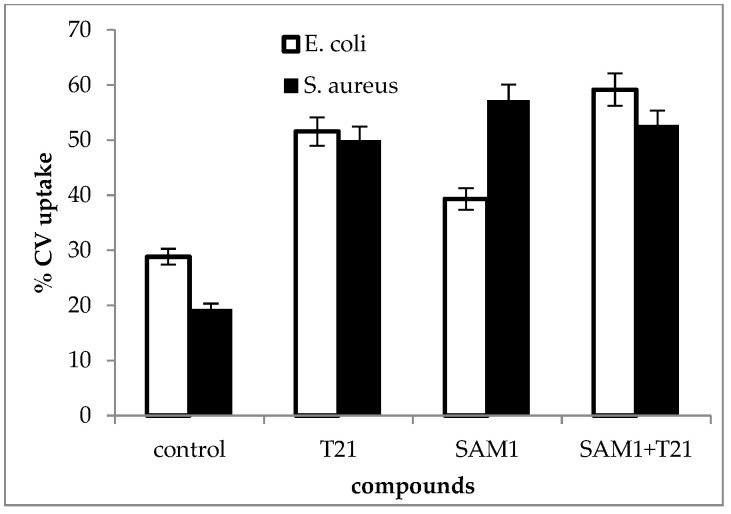
Crystal violet uptake in *S. aureus* ATCC 33952 and *E. coli* ATCC 11229 cultures after treatment with T21, SAM1, and SAM1 + T21 (concentration of compounds and combinations: T21 0.5%; SAM1 1MIC; SAM1 1MIC + T21 0.5%).

In general, an increase in CV uptake was observed after exposure to T21 (*p* < 0.05), SAM1, and T21 + SAM1 (*p* < 0.05) (Figure 8). Although the pattern of dye uptake was more or less quantitatively similar for the two bacteria, some differences were observed.

**Figure 9:**
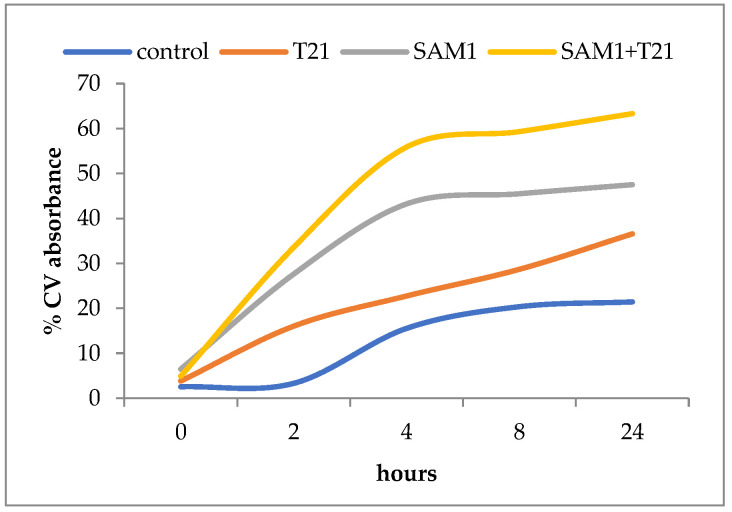
Crystal violet uptake in *E. coli* ATCC 11229 over 24 h after treatment with T21, SAM1, and SAM1 + T21 (concentration of compounds and combinations: T21 0.5%; SAM1 1MIC; SAM1 1MIC + T21 0.5%).

Thus, SAM1 appears to have had a greater effect on membrane permeability in *S. aureus* ATCC 33952 compared to *E. coli* ATCC 11229. Moreover, *E. coli* 11229 seemed more sensitive to the action of T21 than *S. aureus* ATCC 33952. Probably, T21 exerted its effect on the outer membrane of *E. coli* 11229 where it could have easily mobilized a number of components, allowing at the same time the increased intracellular transport of SAM1. By contrast, *S. aureus* ATCC 33952 showed a greater membrane stability, and SAM1 absorption apparently did not benefit from the facilitating effect of T21. An experiment was also performed to understand the dynamics of dye absorption over time in *E. coli* ATCC 11229. The most intense absorption of the dye was recorded for the combination SAM1 + T21 (*p* < 0.05), which suggested a cumulative effect of the two compounds on the CV transport inside the cells (Figure 9).

Unlike the control variant, in which case a lag phase of approximately 2 h was observed, in all the other experimental variants the CV absorption was more intense in the first four hours, after which it slowed down significantly. In the case of T21, the absorption was weaker than for SAM1 and SAM1 + T21, but it seemed to continue at the same rate beyond 24 h of observation.

## 4. Discussion

**Figure 10:**
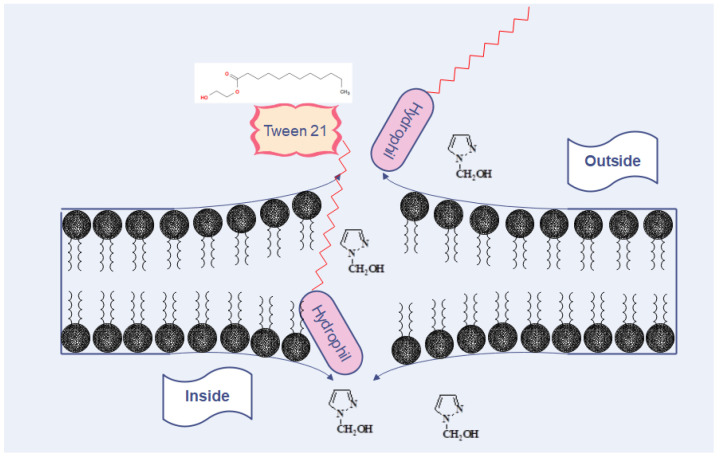
The potential mechanism of the action of T21 affects the influx of pyrazoles to the inside of bacterial cells. T21 interacts and destabilizes the double layer of phospholipids with the creation of “pores”, through which the improved transfer of pyrazoles takes place. In this way, the accumulation of pyrazole leads to the increase in the intracellular concentration and the killing of bacterial cells, a fact that could explain the decrease in the MIC and the improvement of the antimicrobial effect of pyrazoles when combined with T21.

Regarding the MIC, our results were more or less similar to those reported by other authors who found values for pyrazole derivatives ranging from 100 µg to 500 µg/mL [12,32] and 500 to 1000 µg/mL [11]. The presence of T21 had variable effects depending on the bacterial group. The SAM1/T21 combination showed an increase in the inhibitory efficacy in Gram-negative bacteria when compared with SAM1 alone. Moreover, significant changes were recorded in AM1 activity when combined with T21, except for the clinical strains of *Staphylococcus* when the same value was observed for both AM1 and AM1 + T21. It should therefore be noted that the effect of the SAM1/T21 combination depended on the bacterial strain. In Gram-negative bacteria, the presence of T21 decreased the inhibitory concentration of SAM1 but did not affect the activity of AM1 in the *Staphylococcus* clinical strains. Therefore, the effect of T21 depended on the associated compound as well as on the bacterial species. The time-kill experiments revealed that the addition of T21 increased the bactericidal rate of both compounds. In *E. coli*, the cumulative effect was much more obvious, and the bactericidal potential increased from 1 MIC of the compounds alone to 2 MIC and 4 MIC when they were used in combination with T21. As with most pyrazole-containing molecules [33,34,35], AM1 and SAM1 can act by inhibiting bacterial gyrase. Gyrase is the enzyme that controls the normal process of replication and the correct spatial organization of the bacterial chromosome. It is composed of two chains, GyrA and GyrB subunits that are responsible for the producing negative supercoils in DNA during replication [36]. Antimicrobials that target DNA gyrase exhibit their antibacterial activity by two mechanisms, as gyrase inactivation or by interfering with the normal ATP binding at a specific site [37]. Compared to the use of pyrazoles alone, it is likely that the combination of pyrazoles/T21 increases the intracellular concentration of pyrazoles, which leads to the increased inactivation of bacterial gyrase (Figure 10). Overcoming the resistance of pathogenic bacteria to antimicrobials through the use of adjuvants can be achieved by using agents that permeabilize the membrane [38]. Along with other resistance mechanisms, bacteria can change their membrane permeability, and thus, they are able to restrict the penetration of the inhibitory substance inside the cells [38]. Therefore, molecules that increase membrane permeability have the ability to stimulate the penetration of specific antimicrobial compounds. In this case, surfactants can play the role of adjuvants that modulate the transport of antimicrobial substances. They can be used under certain conditions to facilitate the penetration and concentration of antimicrobial compounds inside the pathogen cells. Additional studies are needed to clarify the way in which T21 increases membrane permeability, as well as how AM1 and SAM1 interact with gyrase in Gram-positive and Gram-negative bacteria.

However, the effect of the T21 addition was variable and related to a specific group, either Gram-positive or Gram-negative. The differences observed could be attributed to the ultrastructural and enzymatic particularities of the Gram-positive and Gram-negative groups. As stated earlier, the surfactants can affect the functioning of the cell components as well as the cell metabolism. In Gram-negative bacteria, the inner membrane, outer membrane, and periplasmic space play a critical role in the intracellular transfer of various molecules [23]. It is therefore possible that T21 interacts with the transport function of some factors from the periplasmic space and stimulates the penetration of pyrazoles. Lipases are other factors that could explain, at least in part, the differences observed in pyrazole/T21 inhibitory activity. Both Gram-positive and Gram-negative bacteria synthesize lipases, and so, they can breakdown surfactant molecules and modulate their effect on the bacterial cells [39,40]. In staphylococci, lipase production is common, and they appear to play the role of specific virulence factors. They facilitate the colonization and persistence of pathogens on the skin [41,42]. In Gram-negative bacteria, the surfactants can inhibit the development of biofilms [43,44], but some mutant strains of *Ps. aeruginosa* are able to cleave surfactants and release the fatty acids from their molecules. Lipase could be responsible for cleavage of surfactants and their subsequent inactivation [45]. In this way, bacteria-producing lipases were able to overcome the detrimental effect of surfactants and develop biofilm in their presence. Furthermore, one related compound, polysorbate 80 (T80), appeared to stimulate lipase production in *Burkholderia glumae* [46]. It was also observed that T80 increased the growth rate of planktonic *S. aureus* [47]. Some authors noted that this surfactant could lower the efficacy of some antibacterials, such as rifampicine and isoeugenol [47]. In our experiments, however, an effect of the potentiation of the antibacterial activity of the studied pyrazoles was observed, with a more pronounced effect in the Gram-negative bacteria compared to the Gram-positive ones, due to the ultrastructural peculiarities of the two groups.

The release of macromolecules outside the cells can be seen as a sign of the loss of the normal structure and functionality of the bacterial cells. In *S. aureus* ATCC 33952, a significant increase in 200 nm absorbance above the control was recorded for the T21 + SAM1 combination (Figure 5), with a value that approached the absorbance of lysate. In *E. coli* ATCC 11229, exposure to both SAM1 and T21 + T21 induced a slightly higher absorbance compared to the control. Interestingly, the absorbance value was lower when both bacteria were exposed to T21 (Figure 5), suggesting a protective role of the surfactant on membrane stability. The absorbance at 200 nm indicates the presence of the carbonyl group and peptides [48,49]. It may also indicate the extracellular release of lipopolysaccharide fractions [50] and phospholipids [51]. The absorbance at 260 nm is used to quantify the nucleic acids [52,53], and our experiments suggested that a significant amount of DNA is released outside the cell after the exposure of bacterial cells to pyrazoles and their combinations with T21. The maximum effect was observed in *E. coli* ATCC 11229 when the combination of T21 + SAM1 was associated with high 260 nm absorbance (Figure 6). Most probably, T21 induced changes in the cell membrane and in this way facilitated the intracellular transport of SAM1. In turn, SAM1 led to the structure alteration of some cellular components and to the release of proteins from the cells. Therefore, the association of the two compounds resulted in the decrease in the lethal concentration, a fact reflected by the decrease in the MIC when the two compounds were associated. The measurements performed at 280 nm, which was characteristic for the presence of proteins, especially those with an aromatic ring [54], showed that the increase in absorbance under the influence of SAM1 was more intense in *S. aureus* ATCC 33952 compared to *E. coli* ATCC 11229, suggesting that the outer membrane of Gram-negative bacteria plays an important role in modulating the activity of these two compounds. On the other hand, the compounds and their combinations could act differently on some macromolecules depending on their structure, such as nucleic acids and proteins, a fact illustrated by the differences in the absorbance at 260 nm and 280 nm. The release of intracellular material (nucleic acids, proteins, and peptides) was observed in many cases when the bacteria interacted with antimicrobials, such as antibiotics, essential oils, etc. Many reports showed increased UV absorbance in bacterial cultures when exposed to a large variety of antimicrobial compounds [32,55,56,57]. *S. aureus* exposure to biosurfactants (rhamnolipids) has resulted in the release of intracellular macromolecules and increased UV absorbance [55]. This hypothesis should be viewed with some reservations because unlike T80, which contains oleic acid, T21 is made up of lauric acid. As a result, the degradation of T21 could be achieved by different lipase-like enzymes.

Essential oils also have detrimental effects on the cell membrane and affect its permeability [56]. They were accompanied by increased UV absorbance [57] and the release of cellular material [32]. In conclusion, UV absorbance showed that the exposure of bacterial cells to T21, SAM1, and SAM1 + T21 might cause the release of cell material due to increased membrane permeability. In turn, increased permeability could allow a higher amount of SAM1 to be transported inside the bacterial cells, which in some cases might explain the increased antimicrobial effect of SAM1 when associated with T21. The CV uptake test was commonly used to detect changes in the permeability of the membrane when bacteria were subjected to the action of antimicrobials. Increased permeability and increased absorbance of CV have been observed in numerous experiments that tested a variety of compounds with antimicrobial activity, such as lauric arginate and cinnamon oil [58], coraligin [59], eugenol [60], and essential oils extracted from different sources [32,61]. The above observations may be useful in situations where antimicrobials are used in combination with surfactants in various pharmaceutical formulations. The influence of T21 and its interference with some antimicrobials depends on its concentration as well as on the chemical structure of the antimicrobial agent [62]. In some cases, the antimicrobial effect may be enhanced in the presence of surfactants, as seen in the report of Churchward et al., 2020 [63], which showed that T21 might be useful as a delivery agent for antimicrobials, for example, in ocular antibacterial formulations with capridine.

## 5. Conclusions

The association of T21 with SAM1 and AM1 increased the MIC efficacy of AM1 and SAM1. The time-kill dynamics confirmed the MIC results, and the combination of SAM1 and AM1 with T21 accounted for a significant increase in the killing rate in *S. aureus* and *E coli*. Based on the UV absorbance data and the CV uptake, we assume that the stimulation of the antibacterial activity of pyrazoles when associated with T21 could be assigned to the increase in bacterial membrane permeability. This effect could be used in practical applications to exploit the properties of non-ionic surfactants to increase the antibacterial potential of a wide variety of chemical structures. Carefully chosen surfactants can be valuable adjuvants for combating antimicrobial resistance when the resistance mechanism relies on the selective decrease in bacterial membrane permeability to specific antimicrobials. Compounds similar to T21 could be included in a list of adjuvants and associated with a diversity of antimicrobials to find solutions to overcome antibiotic resistance, at least in some particular cases.

## Figures and Tables

**Table 1:** Bacterial strains tested against SAM1, AM1, and their combination with T21.

Crt. No.	Strain	Phenotypic Characteristics	Abbreviation
1	*Proteus mirabilis*	Clinical strain isolated from urinary tract infection, resistant to ampicillin, aztreonam cefuroxime, imipenem, nitrofurantoin, and tetracycline	PM1
2	*Escherichia coli*	Clinical strain isolated from urinary tract infection, resistant to nalidixic acid, amoxicillin, cotrimoxazole, and cefalotin	EC1
3	*Pseudomonas aeruginosa*	Clinical strain isolated from ear infection, pyocianin production, resistant to aztreonam ciprofloxacin, cefpirome, gentamicin, levofloxacin, minocycline ticarcilin, netilmicin, tobramycin, and pefloxacin	PA1
4	*Staphylococcus aureus*	Clinical strain isolated from skin infection, methicillin-resistant (MRSA), coagulase-positive, haemolytic, resistant to cefoxitin, penicillin, penicillin, ciprofloxacin, erythromycin, clindamycin, and gentamycin	SA1
5	*Staphylococcus aureus*	Clinical strain isolated from skin infection, (MRSA), coagulase-positive, haemolytic, resistant to azithromycin, cloxacillin, ciprofloxacin, co-trimoxazole, chloramphenicol, clindamycin, tetracycline, and gentamycin	SA2
6	*Staphylococcus aureus*	Clinical strain isolated from skin infection, (MRSA), coagulase-positive, haemolytic, resistant to ampicillin ciprofloxacin cefoxitin, clindamycin penicillin, erythromycin gentamycin, and tetracycline	SA3
7	*Escherichia coli* ATCC 11229	Reference strains	ECATTC
8	*Pseudomonas aeruginosa* ATCC 27853	Reference strains	PAATTC
9	*Staphylococcus aureus* ATCC 33952	Reference strains	SAATTC

**Table 2:** MIC for SAM1, AM1, and their combinations with T21 (mean µg/mL).

Crt. No.	Strain	Compounds			
		SAM1	AM1	SAM1 + T21	AM1 + T21
1	PM 1	200	400	100	100
2	PAATCC	200	400	50	200
3	PA 1	400	400	50	200
4	EC1	200	400	50	200
5	ECATTC	200	400	50	200
6	SAATTC	100	200	50	100
7	SA 1	100	200	50	100
8	SA 2	100	200	50	100
9	SA 3	100	200	50	100
Mean		177.77	311.11	55.55	144.44

## Data Availability

All data generated or analyzed during this study are included in this published article.
